# miR-146a-5p Plays an Oncogenic Role in NSCLC via Suppression of TRAF6

**DOI:** 10.3389/fcell.2020.00847

**Published:** 2020-09-02

**Authors:** Xiangdong Liu, Bo Liu, Ruihua Li, Fei Wang, Ning Wang, Maihe Zhang, Yang Bai, Jin Wu, Liping Liu, Dongyu Han, Zhiguang Li, Bin Feng, Guangbiao Zhou, Shujing Wang, Li Zeng, Jian Miao, Yiqun Yao, Bin Liang, Lin Huang, Qi Wang, Yingjie Wu

**Affiliations:** ^1^Institute for Genome Engineered Animal Models of Human Diseases, Dalian Medical University, Dalian, China; ^2^National Center of Genetically Engineered Animal Models for International Research, Dalian Medical University, Dalian, China; ^3^Liaoning Provence Key Lab of Genome Engineered Animal Models, Dalian Medical University, Dalian, China; ^4^Department of Clinical Laboratory, Second Affiliated Hospital of Dalian Medical University, Liaoning, China; ^5^Division of Hepatobiliary and Pancreatic Surgery, Department of General Surgery, Second Affiliated Hospital of Dalian Medical University, Liaoning, China; ^6^Center of Genome and Personalized Medicine, Institute of Cancer Stem Cell, Cancer Center, Dalian Medical University, Dalian, China; ^7^Department of Biotechnology, Dalian Medical University, Dalian, China; ^8^Institute of Zoology, Chinese Academy of Sciences, Beijing, China; ^9^Department of Biochemistry and Molecular Biology, Institute of Glycobiology, Dalian Medical University, Dalian, China; ^10^Department of Thyroid and Breast Surgery, Affiliated Zhongshan Hospital of Dalian University, Dalian, China; ^11^School of Life Sciences, Yunnan University, Kunming, China; ^12^Department of Pathophysiology, College of Basic Medical Sciences, Dalian Medical University, Dalian, China; ^13^Department of Respiratory Medicine, Second Affiliated Hospital of Dalian Medical University, Liaoning, China; ^14^Department of Molecular Pathobiology, New York University College of Dentistry, New York, NY, United States; ^15^Division of Endocrinology, Diabetes and Bone Disease, Department of Medicine, Icahn School of Medicine at Mount Sinai, New York, NY, United States

**Keywords:** apoptosis, miR-146a-5p, migration, non-small cell lung cancer, TRAF6

## Abstract

Non-small cell lung cancer (NSCLC) is the most deadly cancer in the world due to its often delayed diagnosis. Identification of biomarkers with high sensitivity, specificity, and accessibility for early detection, such as circulating microRNAs, is therefore of utmost importance. In the present study, we identified a significantly higher expression of miR-146a-5p in the serum and tissue samples of NSCLC patients than that of the healthy controls. In parallel, miR-146a-5p was also highly expressed in three human NSCLC adenocarcinoma-cell lines (A549, H1299, and H1975) compared to the human bronchial epithelium cell line (HBE). By dual-luciferase reporter assay and manipulation of the expressions of miR-146a-5p and its target gene, tumor necrosis factor receptor-associated factor 6 (TRAF6), we showed that the functional effects of miR-146a-5p on NSCLC cell survival and migration were mediated by direct binding to and suppression of TRAF6. Overexpression of TRAF6 sufficiently reversed miR-146a-5p-induced cancer cell proliferation, migration, and apoptosis resistance. Our data implied that miR-146a-5p/TRAF6/NF-κB-p65 axis could be a promising diagnostic marker and a therapeutic target for NSCLC.

## Introduction

Non-small cell lung cancer (NSCLC) is the most common form of lung cancer and accounts for approximately 80–85% of all lung cancers. Although several comprehensive treatments combining surgery, radiotherapy, and chemotherapy are used for NSCLC, it remains the most deadly cancer type in both genders (Zugazagoitia et al., 2014; Herbst et al., 2018). Thus, a more in-depth understanding of cancer development is urgently needed for early diagnosis and targeted therapy of NSCLC.

MicroRNAs (miRNAs) are a group of small non-coding RNA molecules ofless than 25 nucleotides and present across species from plants, animals to some viruses (Di Leva et al., 2014). miRNAs participate in the regulation of various physiological or pathological processes by direct binding to the 3′ untranslated regions (3′ UTR) of target genes, resulting in mRNA degradation or translational suppression (Polley et al., 2016; Perez-Sanchez et al., 2018). Although the miRNA-guided post-transcriptional control was mainly revealed in the immune system, the evidence is piling up to link it with a great number of other disorders, such as systemic lupus erythematosus (SLE) and common cancers in colon, stomach, ovary, cervical, prostate, and lung (Paik et al., 2011; Hung et al., 2013; Di Leva et al., 2014; Zheng et al., 2015; Polley et al., 2016; Jiang et al., 2017; Yin et al., 2017; Hu et al., 2018; Li et al., 2019). Similar to protein-coding genes, miRNAs can function as either oncogenes or tumor suppressors to be potential prognostic biomarkers (Thai et al., 2010; Jalava et al., 2012; Wang et al., 2016).

As one of the miRNAs implicated in common cancers, miRNA-146a is associated with cancer progression, positively or negatively. It was demonstrated to be expressed significantly higher in the plasma samples of breast cancer patients than those of healthy controls (Kumar et al., 2013; Jiang et al., 2017). There are reports showing the association between increased tissue miR-146a expression and tumor size or tumor recurrence in human oesophageal squamous cell carcinoma (Zhou et al., 2017), and its anti-apoptotic activity in gastric cancer (Xiao et al., 2012). However, a negative impact of miR-146a on cancer development was also reported in prostate cancer (Meng et al., 2020), head and neck squamous cell carcinoma (Lerner et al., 2016), cervical cancer (Hu et al., 2018), and NK/T cell lymphoma (Paik et al., 2011). Not only does the role of miRNA-146a seem to be cancer type-dependent, but also opposing conclusions were reached in studies of NSCLC. One study reported that miR-146a-5p was downregulated in NSCLC tissues (Wu et al., 2014), whereas another study indicated that miR-146a-5p was up-regulated in the serum of NSCLC patients (Wang et al., 2015). Sample size, cancer subtypes and stage, patient background, measurement approaches, and even compensation of miRNA-146b (Paterson and Kriegel, 2017), could be the factors compounding the confusion in publications and await thorough clarification. miR-146a biogenesis and its binding ability to its target genes are also subject to the control of single nucleotide polymorphisms (SNPs), the most common form of DNA variation in the human genome that may effectually contribute to cancer susceptibility (Duan et al., 2007). SNP variance in miR-146a precursor rs2910164 was related to anti-TNF-a treatment in rheumatoid arthritis (RA) patients (Bogunia-Kubik et al., 2016) and also affected phenotypes in breast cancer (BC; Meshkat et al., 2016) and the progression of cervical cancer (Hu et al., 2018). In a female non-smoking lung cancer cohort from Northeast China, a strong association between miR-146a rs2910164 CG or GG genotype and a lower lung cancer risk than the wild-type homozygous CC genotype was found, which might be due to reduced binding of SNP variants to its target gene tumor necrosis factor receptor-associated factor 6 (TRAF6; Yin et al., 2017).

As a signal transducer in the miR-146a-5p/TRAF6/NF-κB axis that fine-tunes immune homeostasis, TRAF6 is a downstream effector of miR-146a-5p, with pleiotropic roles in carcinogenesis, cancer invasion and metastasis (Meng et al., 2012; Zhang X.L. et al., 2014). Via down-regulation of TRAF6, miR-146a-5p suppressed hepatocellular carcinoma (HCC) cell proliferation and invasion *in vitro* and inhibited tumor formation *in vivo* and *in vitro* (Zu et al., 2016). miR-146a was not only overexpressed in cervical cancer and promoted cancer cell proliferation by targeting TRAF6 through NF-κB signaling (Li et al., 2019), but also in oral carcinoma by targeting IRAK1, TRAF6, and NUMB (Hung et al., 2013; Min et al., 2017). miR-146a-5p/TRAF6/NF-κB-p65 axis regulated cell growth and gemcitabine (GEM) chemotherapy sensitivity in pancreatic ductal adenocarcinoma (PDAC; Meng et al., 2020). However, where the expression of TRAF6 in NSCLC is concerned, discrepant conclusions are drawn. In one cohort, TRAF6 expression was higher in the tumor than surrounding tissues in 59.6% of the patients and was inversely related to chemo-sensitivity, but unrelated to cancer stages or overall survival (Liu et al., 2012). In another similar-sized study, a positive correlation was detected between TRAF6 positivity and cancer stage of both NSCLC and SCLC (Zhang X.L. et al., 2014). Over-expression of TRAF6 gene aligned with the amplification of chromosome 11 at band p13 was proposed to constitute the MAPK pathway activation in human lung cancer development (Starczynowski et al., 2011). Whether the murky picture of TRAF6 in NSCLC coincides or has a mechanistic relationship with miR-146a-5p regulation was not analyzed in these reports and is to be addressed here.

In this study, we determined the status of miR-146a-5p in the serum and tissue samples from NSCLC patients and in the NSCLC cell lines, its role in cancer cell proliferation and migration. We identified TRAF6 as the important target gene of miR-146a-5p to promote NSCLC cancer survival.

## Materials and Methods

### Tissue and Serum Samples

Serums from a total of 36 NSCLC cancer patients and 11 healthy controls and 16 NSCLC cancer tissues with corresponding adjacent paracancerous tissue specimens were obtained from the Second Affiliated Hospital of Dalian Medical University (Dalian, China). The healthy control samples were collected from individuals seeking routine physical health examination in the hospital and having negative results in lung cancer screening. All subjects were thoroughly informed in writing about the research procedure and have given consent to participate. The study protocol was approved by the Ethics Committee of Second Affiliated Hospital of Dalian Medical University. During the surgical treatment, all tissues were immediately stored at −80°C until the RNA and protein were later extracted for experiments. In addition, the related information on the clinical samples is shown in Supplementary Figure S1.

### Cell Culture

The human bronchial epithelium cell line (HBE) and NSCLC cell lines A549, H1299, and H1975, were obtained from American Type Culture Collection (ATCC, Manassas, VA, United States). All cell lines were cultured in RPMI-1640 medium (Thermo Fisher Scientific, Waltham, MA, United States) containing 10% fetal bovine serum (FBS; Gemini Bio Products, West Sacramento, CA, United States), 100 IU/ml penicillin and 100 μg/ml streptomycin (Solarbio^®^ Life Sciences, Beijing, China), at 37°C with 5% CO_2_ in a humidified incubator (NuAire, Plymouth, MN, United States).

### Cell Transfection

Cells were plated in 24-well plates or 10 cm dishes. When reached 70–80% confluence, cells were transfected with 100 nM miR-146a-5p mimics, miR-146a-5p inhibitor, siTRAF6 and 500 ng plasmids (GenePharma, Suzhou, China), using Lipofectamine 3000 reagent (Invitrogen, Carlsbad, CA, United States) following the manufacturer’s instruction. All oligonucleotide sequences are listed in Supplementary Table S1.

### RNA Extraction and qRT-PCR

Total RNA was extracted from frozen tissues or cultured cells using RNAiso Plus Reagent (Takara, Dalian, China), according to the manufacturer’s protocols. cDNA was synthesized from mRNA using the TransScript One-Step gDNA Removal and cDNA Synthesis SuperMix (Transgene, Beijing, China), and miRNA reverse transcription was performed using One-Step miRNA cDNA Synthesis Kit (Transgene), following the manufacturer’s instruction. The levels of mRNA or miRNA were quantified by qRT-PCR, using the TransStart Tip Green qPCR SuperMix (Transgene). The endogenous controls for mRNA and miRNA were GAPDH and U6, respectively. The data were calculated using the 2^–ΔΔ*CT*^ method. Primer sequences are listed in Supplementary Table S2.

### Cell Proliferation Assay

Cells were plated in 96-well plates (Corning, New York, United States) at a density of 3 × 10^3^/well and transfected with miRNA mimics/inhibitors or siRNA at about 40% confluency. Cell viability was assessed at 0, 24, 48, and 72 h after transfection by adding 10 μl of Cell Counting Kit-8 (CCK-8; Transgene) reagent and 90 μl non-serum RPMI-1640 into each well and incubation for 30 min. The absorbance at 450 nm was measured using an EMax Plus Microplate Reader (Molecular Devices, San Jose, CA, United States).

### Immunofluorescence Assay

Cells were plated in 24-well plates and grown to about 40% confluence when transfected with miRNA mimics/inhibitors or siRNA for 24 h. Cells were then fixed with 10% neutral buffered formalin (Coolaber, Beijing, China) for 30 min at room temperature. The fixed cells were incubated with a primary rabbit antibody against ki-67 (Proteintech, Beijing, China) overnight at 4°C. After incubation with Dylight 594 conjugated goat anti-rabbit IgG secondary antibody (Abbkine, Redlands, CA, United States) for 2 h at room temperature, the 4, 6-Diamidino-2-phenylindole (DAPI; Sigma Corporation of America, Ronkonkoma, NY, United States) at 1:2000 was added for 30 min. The cells were visualized using a fluorescence microscope (Nikon ECLIPSE Ni-E, Tokyo, Japan).

### Transwell Migration Assay

Twenty-four-well plates and 8-μm Transwell cell culture chambers (Promega Corporation, Madison, WI, United States) were utilized for transwell assay. Briefly, 1 × 10^5^ cells in 200 μl serum-free medium were placed into the upper chamber. RPMI-1640 medium containing 10% FBS (Gemini Bio Products) was added into the lower chamber as a chemo-attractant. Cells that did not migrate after 24 h in the upper part of the filters were removed with a cotton swab. The membranes were next fixed by 10% neutral buffered formalin (Coolaber) for 30 min and stained with 0.5% crystal violet (Beyotime Biotechnology, Shanghai, China) for 10 min. The membranes were washed thoroughly with 1 × PBS and dissolved in 500 μl 33% acetic acid. The migrated cells were detected at 200 × or 100 × magnification in at least six different fields of each filter and the absorbance was measured at 570 nm.

### Wound Healing Assay

Cells were placed into six-well plates at a density of 5 × 10^4^/well. When the cells were 90% confluent, a straight wound line was scraped with a 10 μl pipette tip in each well. Pictures were snapped before and after the subsequent 24 h incubation, using a phase-contrast microscope (Nikon ECLIPSE TS100). The cell migration rate in the scratch areas was quantified by ImageJ software.

### Apoptosis Analysis

After transfection with miR-146a-5p mimics, miR-146a-5p inhibitor, siTRAF6 and TRAF6-overexpression plasmids or their negative controls for 48 h, cells were resuspended in 200 μl Binding Buffer (Solarbio^®^ Life Sciences, Beijing, China) at 5 × 10^5^/ml. Cell suspension was incubated in 10 μl Annexin V-FITC (Solarbio^®^ Life Sciences) and 10 μl propidium iodide (PI; Solarbio^®^ Life Sciences) for 15 min in the dark. The reaction was terminated by adding 300 μl Binding Buffer and analyzed by flow cytometry (BD FACS Canto II, Franklin Lakes, NJ, United States). Cells stained positive for FITC-Annexin were considered apoptotic. TRAF6 ORF was cloned into pEX-3 vector (GenePharma, Shanghai) at XhoI/EcoRI sites under the control of CMV promoter. The complete plasmid map and sequence are available upon request.

### Dual-Luciferase Reporter Assay

To demonstrate the direct interaction between miR-146a-5p and TRAF6, the 3′ UTR sequence of TRAF6 mRNA containing the putative miR-146a-5p binding sites was synthesized by Sangon Biotech (Shanghai, China), and sub-cloned to downstream of the firefly reporter gene in the pmirGLO vector (Promega, Madison, WI, United States), which was named pmirGLO-TRAF6-WT. Mutant TRAF6 3′ UTR, containing a mutated binding site for miR-146a-5p, was cloned into pmiRGLO and named pmiRGLO-TRAF6-MUT. The oligonucleotide sequences are listed in Supplementary Table S1. For the dual luciferase reporter assay, cells were cultured in a 96-well plate and co-transfected with 100 nM miR-146a-5p mimics and 200 ng pmirGLO-TRAF6-WT reporter plasmid or pmiRGLO-TRAF6-MUT vector using Lipofectamine 3000 (Invitrogen). The luciferase activity was measured 48 h after transfection, using the Dual-Glo Luciferase Assay System (Promega) and assayed with Multimode Plate Reader (Coring, NY, United States). The relative transcriptional activities were expressed as the fold change above the vehicle control in luciferase activity after normalization to renilla luciferase activity.

### Western Blot

At 24 h post-transfection, total proteins were extracted from the cells using RIPA buffer (Solarbio^®^ Life Sciences), and quantified with a Protein BCA Assay Kit (Beyotime Biotechnology). The protein lysates were separated by sodium dodecy1 sulfate-polyacrylamide gel electrophoresis (SDS-PAGE) and transferred to polyvinylidene difluoride (PVDF) membrane (PALL, Ann Arbor, MI, United States). After blocking in 5% skimmed milk (BD, San Jose, CA, United States) at room temperature for 2 h, the membranes were blotted with specific primary antibodies overnight at 4°C. The membranes were probed with secondary antibodies conjugated to horseradish peroxidase (HRP; Abbkine) after washing with 1 × TBST. The protein bands were detected using ECL solution (Abbkine) and imaged with BIO-RAD system (Bio-Rad Laboratories, Stuttgart, Germany). Primary antibody information is listed in Supplementary Table S3.

### Statistical Analysis

All numerical data were presented as mean ± SD. Statistical analysis was carried out with Student’s *t*-test in GraphPad Prism6. Data were considered to be significantly or very significantly different when *p <* 0.05 (marked with ^∗^) or *p <* 0.01 (marked with ^∗∗^), respectively.

## Results

### miR-146a-5p Is Overexpressed in NSCLC Serum, Tissues and NSCLC Cell Lines

**FIGURE 1:**
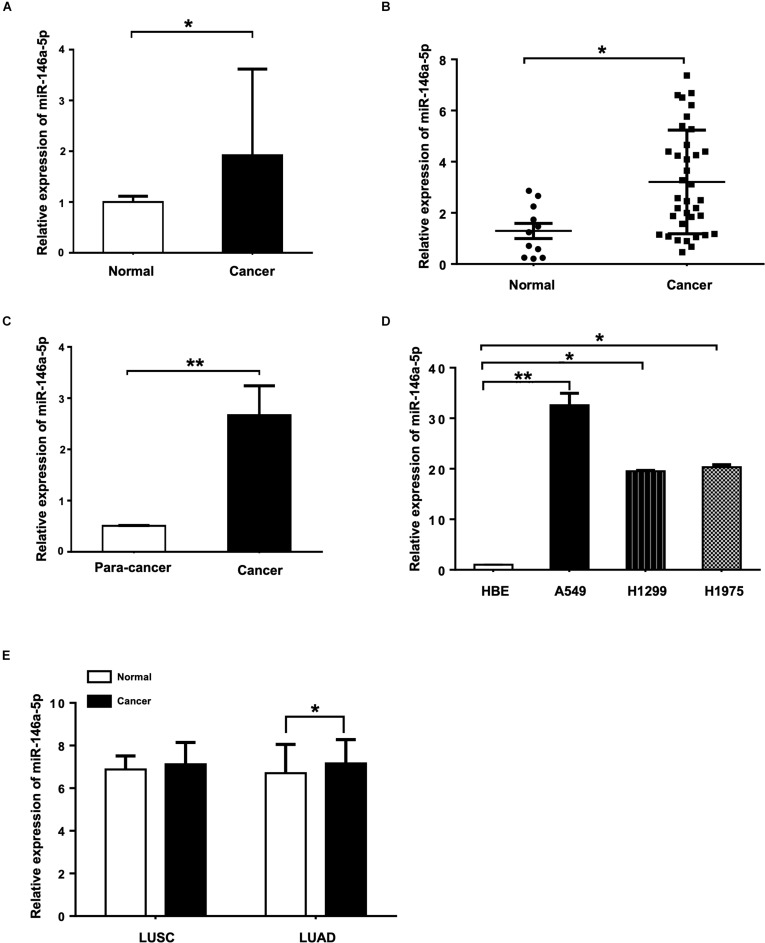
miR-146a-5p expression is up-regulated in NSCLC tissues, serum samples and cell lines. **(A)** Expression of miR-146a-5p in NSCLC patient and normal serum samples measured by miRNA Solexa sequencing. **(B)** qRT-PCR validation of the result from **(A)**. **(C)** Expression of miR-146a-5p in NSCLC cancer and corresponding para-cancer tissues determined by qRT-PCR. **(D)** Expression of miR-146a-5p in three NSCLC cell lines (A549, H1299 and H1975) and human bronchial epithelium cell line (HBE) determined by qRT-PCR. **(E)** Expression of miR-146a-5p from TCGA. LUSC: lung squamous cell carcinoma, LUAD: lung adenocarcinoma.

To determine the miR-146a-5p expression level in the serum of NSCLC patients and the healthy controls, miRNA Solexa sequencing (Figure 1A) and qRT-PCR (Figure 1B) were conducted. Both results showed that the expression of miR-146a-5p was significantly higher in NSCLC serum than in the normal serum samples. Measured by qRT-PCR, miR-146a-5p gene expression in NSCLC cancer tissues had a five-fold increase compared to the corresponding paracancerous tissues (Figure 1C), and was significantly overexpressed in three NSCLC cell lines (A549, H1299, and H1975) when compared with the human bronchial epithelium cell line (HBE; Figure 1D). miR-146a-5p was overexpressed in lung squamous cell carcinoma (LUSC) and lung adenocarcinoma (LUAD) from TCGA database (Figure 1E). These results suggested a potential involvement of miR-146a-5p in the initiation and tumorigenesis of NSCLC. A549 and H1299 cells were chosen for subsequent overexpression and down-regulation experiments, as they had the lowest and highest miR-146-5p expression among the tested NSCLC cell lines, respectively.

### Overexpression of miR-146a-5p Promotes the Proliferation and Migration of A549 Cells

**FIGURE 2:**
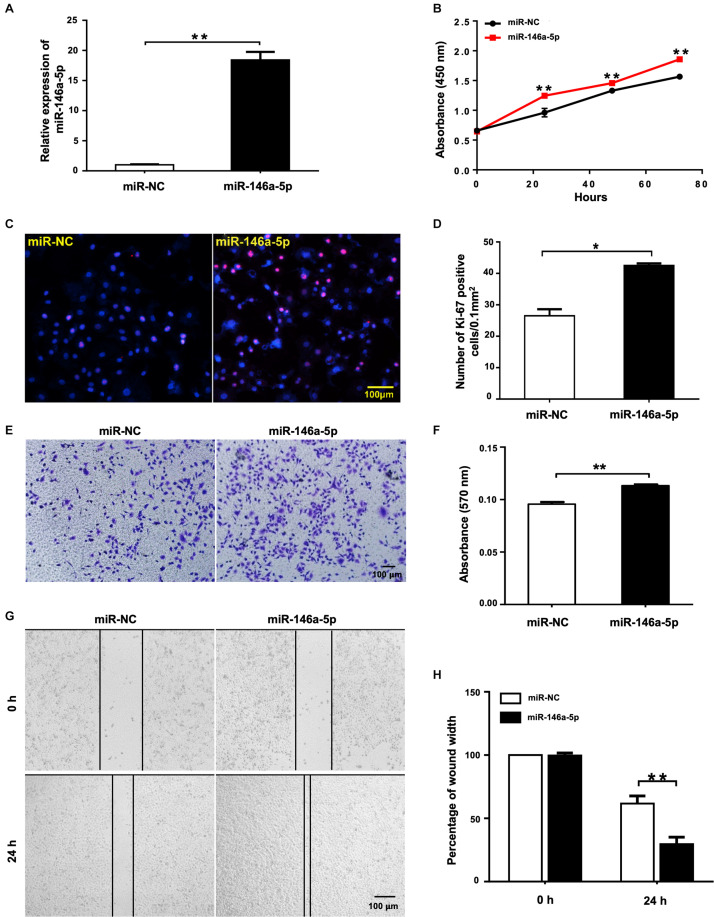
miR-146a-5p overexpression promotes cell proliferation and migration in NSCLC cells. Cell proliferation and migration measurement of A549 cells at 24 h post transfection of miR-146a-5p mimic or miR-NC. **(A)** The mRNA expression of miR-146a-5p measured by qRT-PCR. **(B)** Cell proliferation examined by CCK-8 assay. **(C)** Cell proliferation measured by Ki-67 staining. **(D)** Quantification of the Ki-67 staining assay result of **(C)**. **(E)** Migration ability measured by transwell assay. **(F)** Quantification of the transwell assay result of **(E)**. **(G)** Migration ability detected by wound-healing assay. **(H)** Quantification of the wound-healing assay result of **(G)**.

To evaluate the biological effects of miR-146a-5p on NSCLC cell lines, miR-146a-5p mimic (miR-146a-5p) or its corresponding negative control (miR-NC) was transfected into A549 cells. The transfection efficiency was confirmed by significant up-regulation of miR-146a-5p gene expression in A549 cells after transfection (Figure 2A). In the meantime, cell proliferation of A549 cells, as determined by CCK-8 assay, was enhanced by miR-146a-5p overexpression (Figure 2B). Since the cells sustained a stable higher rate of proliferation within 3 days after transfection, the time point to harvest the cells for other cellular assays was set at 24 h post-transfection. The indication of active cell proliferation promoted by miR-146a-5p was reinforced by strong immunofluorescence staining of Ki-67, a validated index of lung cancer malignancy (Warth et al., 2014; Figures 2C,D). The transwell assay result (Figures 2E,F) and wound healing assay (Figures 2G,H) showed that the cells transfected with miR-146a-5p mimic had a higher migratory capacity.

### Suppression of miR-146a-5p Inhibits the Proliferation and Migration of H1299 Cells

**FIGURE 3:**
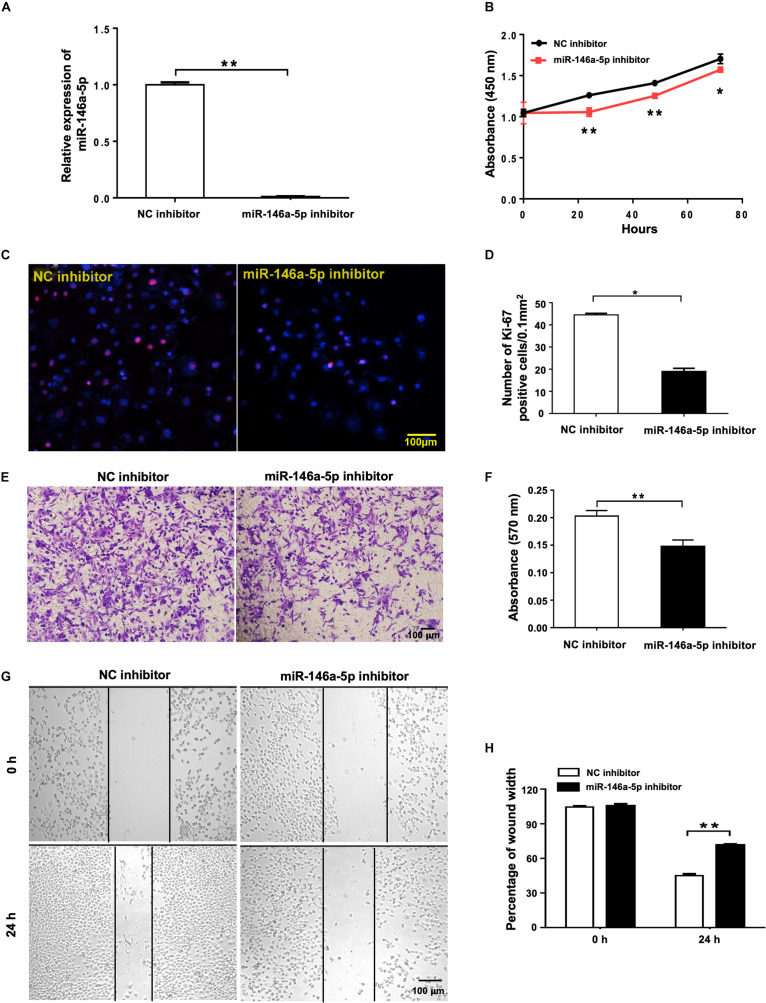
miR-146a-5p inhibitor suppresses cell proliferation and migration in NSCLC cells. Cell proliferation and migration measurement of H1299 cells at 24 h post transfection of miR-146a-5p inhibitor or NC inhibitor. **(A)** The mRNA expression of miR-146a-5p measured by qRT-PCR. **(B)** Cell proliferation examined by CCK-8 assay. **(C)** Cell proliferation measured by Ki-67 staining. **(D)** Quantification of the Ki-67 staining assay result of **(C)**. **(E)** Migration ability measured by transwell assay. **(F)** Quantification of the transwell assay result of **(E)**. **(G)** Migration ability detected by wound-healing assay. **(H)** Quantification of the wound-healing assay result of **(G)**.

To further study the effects of miR-146a-5p on NSCLC cell growth from another angle, miR-146a-5p inhibitor or its corresponding control (NC inhibitor) was transfected into H1299 cells. In contrast to miR-146a-5p overexpression, suppression of miR-146a-5p in H1299 cells by miR-146a-5p inhibitor (Figure 3A) led to reduced cell proliferation, shown by both CCK-8 assay (Figure 3B) and Ki-67 staining (Figures 3C,D). Also, the transwell assay (Figures 3E,F) and wound healing assay (Figures 3G,H) concomitantly displayed a reduced migratory capacity of the cells transfected with miR-146a-5p inhibitor. Taken together, loss- and gain-of-function of miR-146a-5p resulted in an overt reversal of the cancer cell behavior.

### miR-146a-5p Inhibits Apoptosis of NSCLC Cells

**FIGURE 4:**
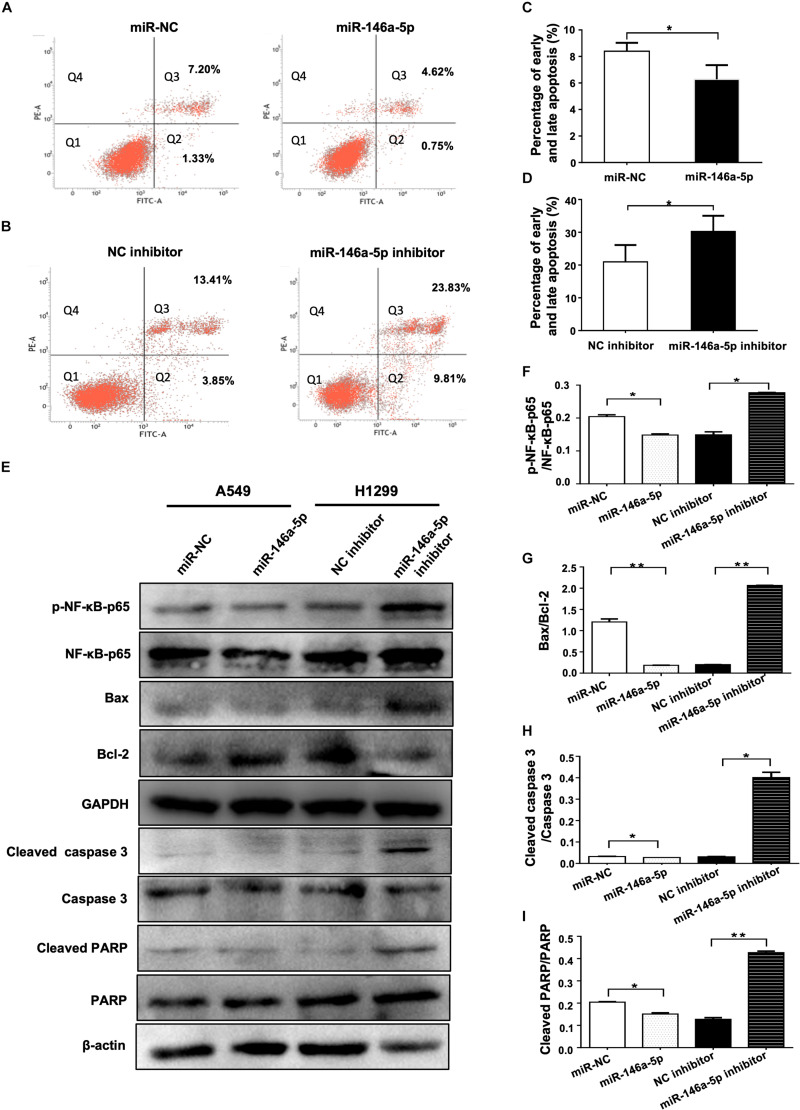
miR-146a-5p inhibits apoptosis in NSCLC cells. **(A)** Cell death determined in A549 cells at 48 h after transfection of miR-146a-5p mimic (miR-146a-5p) or miR-NC, stained with propidium iodide (PI) and annexin-FITC. Q1: Live cells; Q2: early apoptotic cells; Q3: late apoptotic cells; Q4: necroptotic cells. **(B)** Apoptosis in H1299 cells at 48 h after transfection of miR-146a-5p inhibitor or NC inhibitor by FACS as in **(A)**. **(C, D)** Quantification of the early and late apoptosis of **(A, B)**. **(E)** Level of NF-κB-p65(pS536), NF-κB-p65, Bax, Bcl-2, cleaved Caspase 3, Caspase 3, cleave PARP and PARP detected by Western blot in A549 cells and H1299 cells at 48 h post transfection of miR-146a-5p mimic or miR-146a-5p inhibitors, respectively. GAPDH or β-actin used as loading controls. **(F)**. Quantification of pNF-κB-p65(pS536)/NF-κB-p65 ratio as in **(E)**. **(G)** Quantification of Bax/Bcl2 ratio as in **(E)**. **(H)** Quantification of cleaved Caspase 3/Caspase 3 ratio as in **(E)**. **(I)** Quantification of cleaved PARP/PARP ratio as in **(E)**.

As a strategy of advantageous survival, evading apoptosis is one of the major hallmarks of cancer coined by Hanahan and Weinberg (2000). The influence of miR-146a-5p on apoptosis of NSCLC was examined in A549 and H1299 cells transfected with miR-146a-5p mimic (miR-146a-5p) or miR-146a-5p inhibitor, respectively. Flow cytometric analysis demonstrated that the up-regulation of miR-146a-5p resulted in a lower percentage of apoptotic A549 cells (Figures 4A,C) and down-regulation of miR-146a-5p resulted in a higher percentage of apoptosis in H1299 cells (Figures 4B,D). Western blot was used to determine the apoptosis-related genes Bcl-2 and Bax. The Bax/Bcl-2 ratio, which predisposes cell susceptibility to apoptosis (Jaeschke et al., 2018), was decreased by miR-146a-5p overexpression and increased by miR-146a-5p inhibition (Figures 4E,G). Meanwhile, Western blot data showed that miR-146a-5p decreased the phosphorylation level of NF-κB-p65 (pS536; Figures 4E,F) that is required for NF-κB nuclear translocation and its transcriptional activity, implying miR-146a-5p to be a negative regulator of NF-κB signaling pathway. At the same time, miR-146a-5p decreased the level of cleaved caspase 3 and cleaved PARP (Figures 4E,H–I), indicating that miR-146a-5p conferred resistance to apoptosis in NSCLC cells.

### miR-146a-5p Directly Targets TRAF6 in NSCLC Cells

**FIGURE 5:**
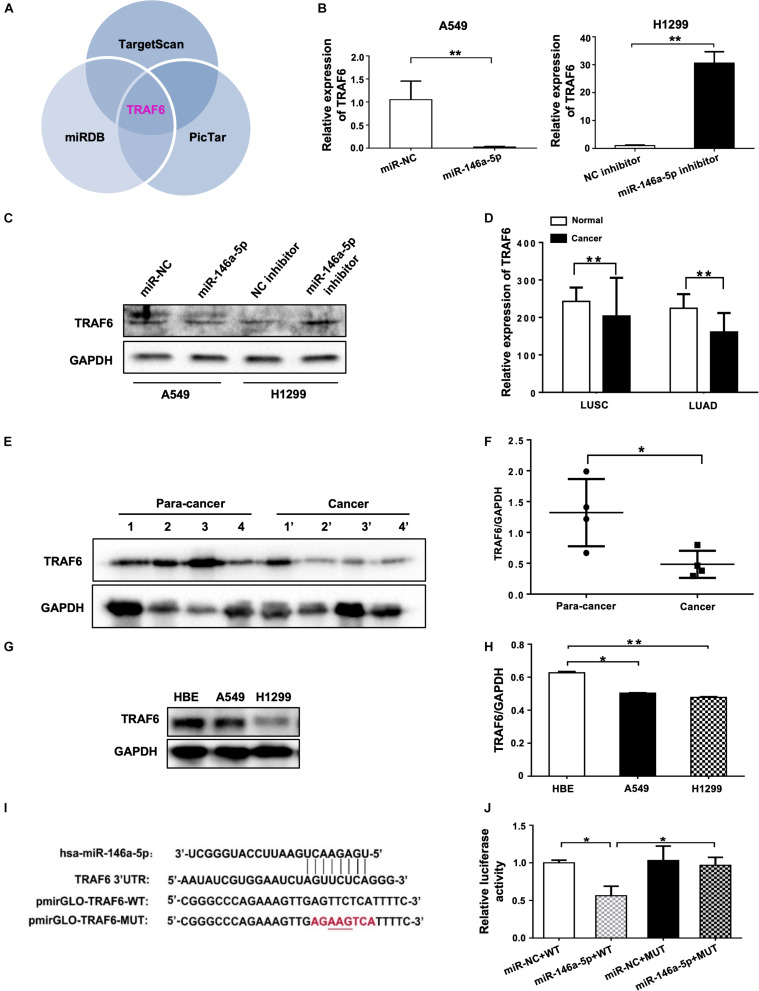
miR-146a-5p directly targets TRAF6 in NSCLC Cells. **(A)** TRAF6 is one of miR-146a-5p targets predicted by TargetScan, miRDB and PicTar databases. Expression of TRAF6 mRNA **(B)** and protein **(C)** in A549 cells or H1299 cells at 24 h transfection with miR-146a-5p mimic, or miR-146a-5p inhibitor respectively. **(D)** Expression of TRAF6 from TCGA, LUSC: lung squamous cell carcinoma, LUAD: lung adenocarcinoma. **(E)** Differential TRAF6 protein levels in NSCLC tissues and the paired adjacent normal tissues. **(F)** Quantitation of WB data as in **(E)**. **(G)** TRAF6 levels in two NSCLC cell lines and HBE cell line. **(H)** Quantitation of WB data as in **(G)**. **(I)** miR-146a-5p binding site within the 3’UTR of human TRAF6 mRNA predicted by TargetScan. Luciferase reporter construct only contains the 7 bp seed sequence and with the center 3 bp mutated in the mutant construct. **(J)** The relative luciferase activity in A549 cells co-transfected with miR-146a-5p mimic WT and MUT luciferase reporter (24 h post transfection).

Using three different databases including miRDB, TargetScan, and PicTar, we identified TRAF6 as one of the targets in the intersection between the groups of predicted targets of miR-146a-5p (Figure 5A). In comparison to the negative control, the mRNA expression and protein level of TRAF6 were decreased in miR-146a-5p mimic (miR-146a-5p)-transfected A549 cells (Figures 5B,C). Consistently, miR-146a-5p inhibitor treatment led to a significant up-regulation of TRAF6 at mRNA and protein levels in H1299 cells (Figures 5B,C). The correlated expression of TRAF6 in NSCLC was next investigated by mining the clinical data from TCGA^1^. A lower expression of TRAF6 was revealed in both lung squamous cell carcinoma (LUSC) and lung adenocarcinoma (LUAD) than in adjacent normal lung tissues (Figure 5D and Supplementary Figures S2A–C). The TRAF6 protein levels were decreased in human NSCLC cancer tissues compared with the paired paracancerous tissues (Figures 5E,F), and were lower in NSCLC cell lines than in the human bronchial epithelium cell line (HBE; Figures 5G,H). According to the miRNA databases, a putative miR-146a-5p binding site located in the 3′ UTR of TRAF6 mRNA was identified. To test whether the TRAF6 gene transcription is directly hindered by miR-146a-5p, the 3′ UTR of TRAF6 sequence that includes the binding site of miR-146a-5p was cloned into the pmiR-GLO plasmids (a dual-luciferase reporter), named pmiRGLO-TRAF6-WT. As control of binding specificity, a pmiRGLO-TRAF6-MUT was constructed by replacement of the binding site with a 3′ UTR mutation (Figure 5I). The pmiRGLO-TRAF6-WT or pmiRGLO-TRAF6-MUT were co-transfected with either miR-146a-5p mimic (miR-146a-5p) or miR-NC into A549 cells. A remarkable reduction of the relative luciferase activity was only observed in cells co-transfected with miR-146a-5p and pmiRGLO-TRAF6-WT, not in cells co-transfected with other combinations (Figure 5J), suggesting that the 3′ UTR sequence of TRAF6 is indeed a direct target gene of miR-146-5p.

### Knockdown of TRAF6 Promotes the Proliferation and Migration, and Inhibits Apoptosis of NSCLC Cells

**FIGURE 6:**
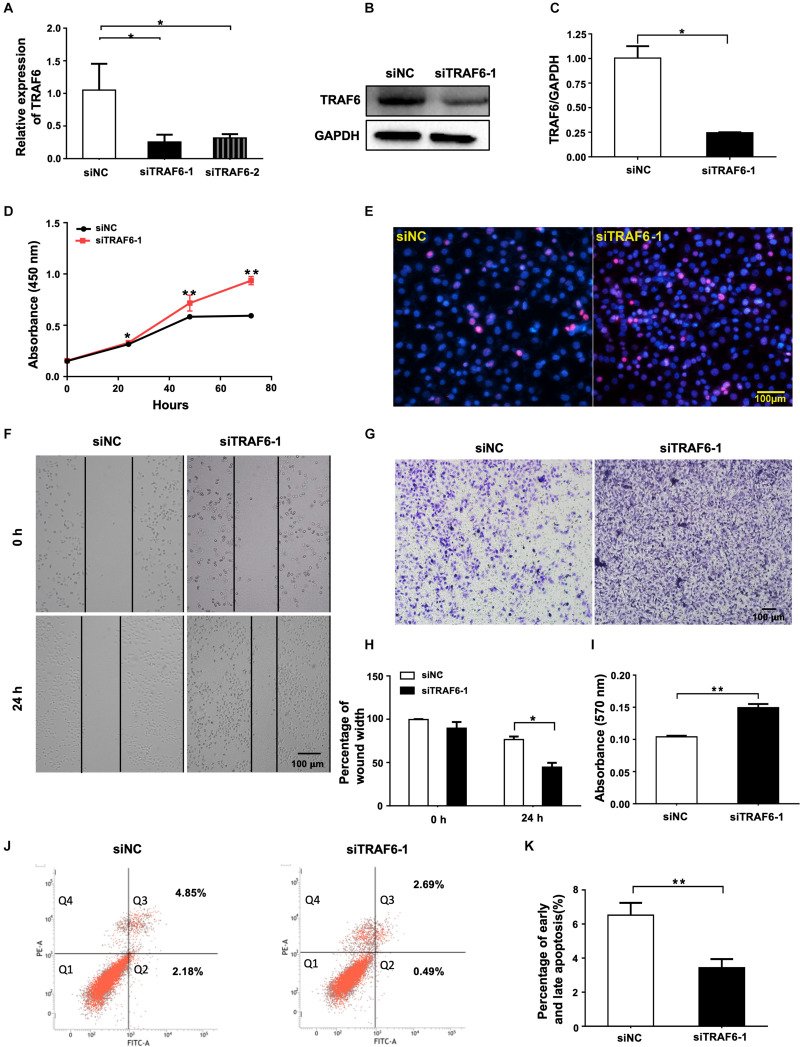
TRAF6 silencing promotes cell proliferation and migration but inhibits apoptosis of NSCLC cells. **(A)** mRNA expression of TRAF6 measured by qRT-PCR. **(B)** The protein level of TRAF6. **(C)** Quantitation of the WB data as in **(B)**. **(D)** Cell proliferation examined by CCK-8 assay. **(E)** Cell proliferation measured by Ki-67 staining. **(F)** Migration ability measured by wound-healing assay. **(G)** Migration ability detected by transwell assay. **(H)** Quantification of the wound-healing assay result as in **(F)**. **(I)** Quantification of the transwell assay result as in **(G)**. **(J)** Early (Q2) and late apoptosis (Q3) determined by flow cytometry analysis. **(K)** Quantification of the FACS data as in **(J)**. All experiments were performed in A549 cells transfected with siRNA for 24 h except in **(B,J)** for 48 h.

To demonstrate the importance of TRAF6 in mediating the pro-survival effects of miR-146a-5p in NSCLC cells, two TRAF6 siRNAs targeting different regions, siTRAF6-1 and -2, and the scrambled control (siNC) were transfected into A549 cells. qRT-PCR results showed that TRAF6 gene was significantly silenced by 75.66 and 69.86% by siTRAF6-1 and -2, respectively (Figure 6A). siTRAF6-1 was used for further experiments because of its higher silencing capacity. The efficiency of TRAF6 silencing by siTRAF6-1 was also confirmed by decreased TRAF6 protein level as shown in Western blot (Figures 6B,C). In agreement with our hypothesis, the above-observed effects of miR-146a-5p overexpression in A549 cells were recapitulated by TRAF6 knockdown, encompassing enhanced cell proliferation (Figures 6D,E), higher migratory capacity (Figures 6F–I) and a lower percentage of apoptotic cells (Figures 6J,K).

### Overexpression of TRAF6 Inhibits the Proliferation and Migration, and Promotes Apoptosis of NSCLC Cells

**FIGURE 7:**
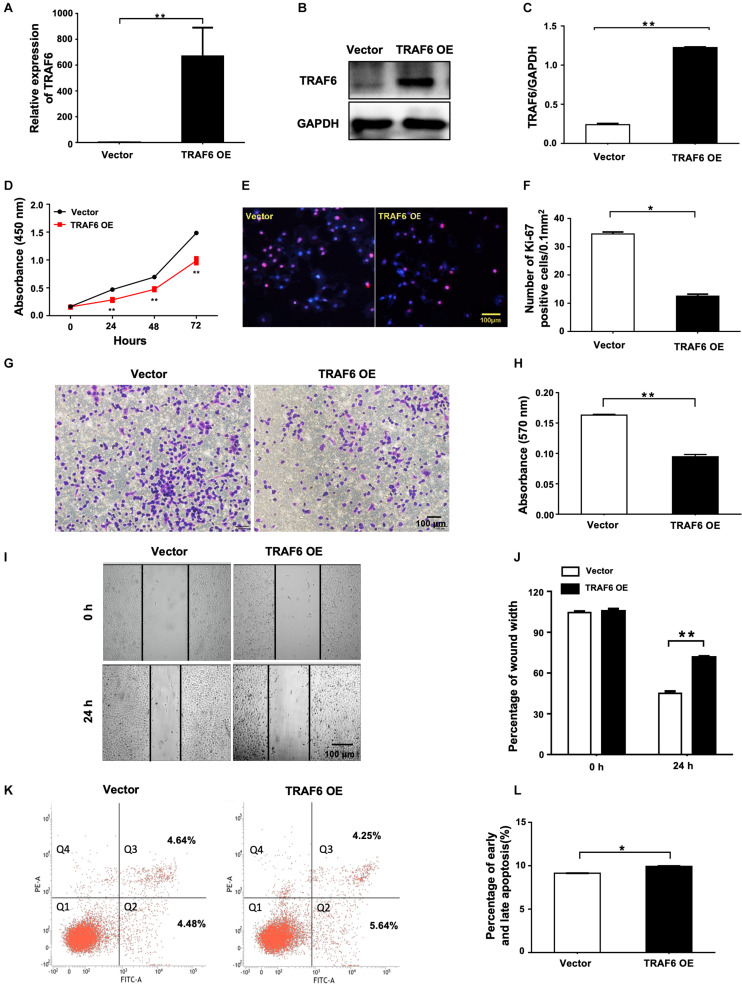
Overexpression of TRAF6 inhibits cell proliferation and migration but promotes apoptosis of NSCLC cells. **(A)** The expression of TRAF6 examined by qPCR. **(B)** The protein level of TRAF6. **(C)** Quantitation of the WB data as in **(B)**. **(D)** Cell proliferation examined by CCK-8 assay. **(E)** Cell proliferation measured by Ki-67 staining. **(F)** Quantification of the Ki-67 staining assay result as in **(E)**. **(G)** Migration ability detected by transwell assay. **(H)** Quantification of the transwell assay result as in **(G)**. **(I)** Migration ability measured by wound-healing assay. **(J)** Quantification of the wound-healing assay result as in **(I)**. **(K)** Early (Q2) and late apoptosis (Q3) measured by flow cytometry analysis. **(L)** Quantification of the FACS data as in **(K)**. All experiments were performed in H1299 cells transfected with TRAF6 overexpression construct for 24 h except in **(K)** for 48 h. TRAF6 OE: overexpression vector of TRAF6.

To investigate the effects of TRAF6 in NSCLC cells, the TRAF6 overexpression (TRAF6 OE) and control (empty vector) were transfected into H1299 cells. TRAF6 was significantly overexpressed at both mRNA (Figure 7A) and protein level (Figures 7B,C). In cells with TRAF6 overexpression, inhibited cell proliferation was confirmed by CCK-8 assay (Figure 7D) and ki-67 staining (Figures 7E,F), while impeded cell migration was detected by transwell (Figures 7G,H) and wound healing assay (Figures 7I,J). However, the percentage of apoptosis in TRAF6-overexpressing H1299 cells was higher than in the control, albeit to a lesser extent (Figures 7K,L).

### Overexpression of TRAF6 Reverses the Malignant Behavior of NSCLC Cells Potentiated by miR-146a-5p Overexpression

**FIGURE 8:**
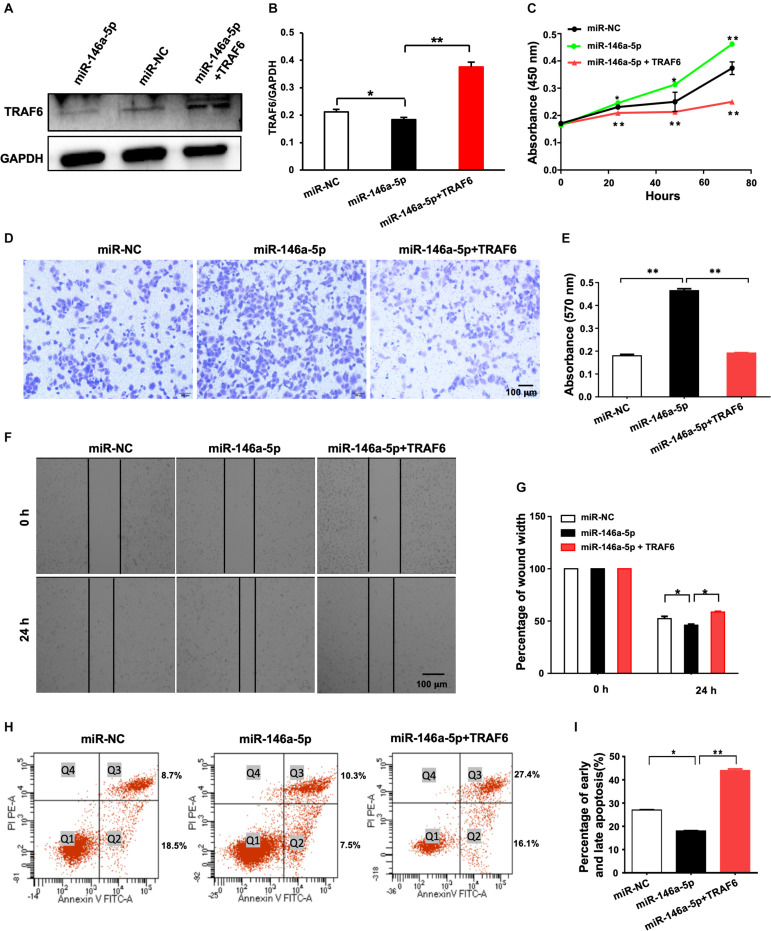
Overexpression of TRAF6 reverses the oncogenic effects of miR-146a-5p in NSCLC cells. **(A)** The protein level of TRAF6. **(B)** Quantitation of the WB data as in **(A)**. **(C)** Cell proliferation examined by CCK-8 assay. **(D)** Migration ability detected by transwell assay. **(E)** Quantification of the transwell assay result as in **(D)**. **(F)** Migration ability measured by wound-healing assay. **(G)** Quantification of the wound-healing assay result as in **(F)**. **(H)** Early (Q2) and late apoptosis (Q3) measured by flow cytometry analysis. **(I)** Quantification of the FACS data as in **(H)**. All experiments were performed in H1299 cells.

A rescue experiment was designed to determine whether restoration of TRAF6 expression in miR-146a-5p overexpression cells is enough to reverse the oncogenic effects of miR-146a-5p. H1299 cells were parallelly transfected with miR-NC, miR-146a-5p mimic (miR-146a-5p) or co-transfected with miR-146a-5p mimic plus TRAF6 overexpression plasmid (miR-146a-5p+TRAF6), respectively. As expected, the examined cancer-specific characteristics of the cells caused by miR-146-5p overexpression were all reversed by TRAF6 plasmid co-transfection. The reduced protein level of TRAF6 by miR-146-5p was fully recovered by the ectopic expression of TRAF6 (Figures 8A,B). The ability of miR-146a-5p to enhance proliferation (Figure 8C), promote migration (Figures 8D–G) and inhibit apoptosis (Figures 8H,I) in H1299 cells was fully reversed by forced expression of TRAF6.

## Discussion

By mining the three online miRNA target prediction databases, we found that TRAF6 is one of the overlaps among all intersecting areas, suggesting it is a favorable target of miR-146a-5p. Direct negative regulation of TRAF6 by miR-146a-5p in NSCLC was further validated by dual-luciferase reporter assay in the current study. Our Solexa sequencing data demonstrated a significantly elevated serum miR-146a-5p expression in NSCLC patients (*n* = 36), with the concurrence of reduced TRAF6 levels at mRNA and protein levels (Figures 5D–H). By manipulating miR-146a-5p or/and TRAF6 levels in NSCLC cell lines, we found that (1) Overexpression of miR-146a-5p resulted in cell proliferation and reduced TRAF6 expression; (2) Inhibition of miR-146a-5p decreased cancerous cellular behavior, accompanied by higher TRAF6 levels; (3) TRAF6 gene silencing by siRNA recapitulate the outcome of miR-146a-5p over-expression; (4) Forced expression of TRAF6 in conjunction with miR-146a-5p overexpression sufficed to revoke the oncogenic effects of miR-146a-5p. These findings confirm that miR-146a-5p plays an oncogenic role in NSCLC cells via direct suppression of TRAF6.

TRAF6 is indispensable in maintaining a balanced immune function and deletion of TRAF6 in mice is lethal as a result of disrupted development and anatomy of the immune system (Starczynowski et al., 2011; Dou et al., 2018). The miR-146a-5p-TRAF6 axis has been proposed to be a key regulator in immune cell activity, hematopoiesis and malignant transformation, the latter involving chronic inflammation induced by dysregulation of miR-146a (Magilnick et al., 2017). The important roles of TRAF6 in immunity (Dou et al., 2018) and tumorigenesis (Yao et al., 2013) are exhibited in the regulation of NF-κB signal pathway (Starczynowski et al., 2011; Wang et al., 2012). It has been reported that miR-146a could negatively regulate particulate matter (PM) – induced inflammation via inhibiting the IRAK1/TRAF6 expression and subsequently prevent the nuclear translocation of p65 in BEAS-2B cells (Liu et al., 2018). But the evidence for regulation of TRAF6 by miRNAs outside the immune system is scarce. TRAF6 was reported to be a target of miR-146a in papillary carcinoma (Cong et al., 2015) and HCC (Zu et al., 2016).

Except for a few confirmed onco-miRs such as miR-21-5p, the role of many miRNAs in cancers displays an inconsistency that constitutes a great hindrance to their translation into diagnostic or prognostic biomarkers of cancer. As emphasized earlier, the clinical implication of miR-146a-5p in NSCLC is polarized to two opposite conclusions. The discrepancies have multiple origins: tumor-intrinsic factors including intratumor heterogeneity, cancer stage and subtypes; cohort factors including tissue/blood sampling, prior treatment, comorbidities, age, sex, and SNP variance; and technical factors including the selection of methods, cell lines and reference genes for RT-PCR normalization, etc. (Condrat et al., 2020). Our data are in agreement with the report from Wang et al. (2015) who found that miR-146a-5p was up-regulated in the serum of NSCLC patients.

However, opposing effects were documented by some other studies. Using different NSCLC cell lines (H358, H1650, and H1975), Chen et al. (2013) found that miR-146a-5p inhibited cell proliferation and induced apoptosis by targeting epidermal growth factor receptor (EGFR) signal pathway. But they did not compare baseline miR-146a-5p levels between NSCLC and normal cells. Besides, they performed transfection of miR-146a-5p mimic/inhibitor daily for 10 days (Chen et al., 2013), in contrast to most studies including ours with 24 or 48 h transfection. Wu et al. (2014) reported that while the stage III cancerous NSCLC tissues or tissues with lymph node metastasis (LNM) displayed lower miR-146a-5p levels than adjacent normal tissues, the majority (over 70%) of the samples at stage I or without LNM had up-regulated miR-146a-5p. Our Solexa sequencing results showed that miR-146a-5p was increased in stages I, II, and III, but indeed decreased in stage IV. Interestingly, one report showed that miR-146a-5p was a lymphatic metastasis-related miRNA in lung adenocarcinoma (Yan et al., 2017). Li et al. (2016) demonstrated reduced miR-146a-5p expression in a few NSCLC cell lines and its suppression of cell proliferation by targeting CCND1 and CCND2. But they also reported only 8 out of 30 clinical cancerous tissues had lowered miR146 levels, which was associated with tumor size > 3 cm (Li et al., 2016). Considering the involvement of miR-146a-5p in various biological processes and pathways (Li et al., 2016), especially inflammatory response, these reports raise the curiosityof temporal dynamics of miR-146a-5p regulation or a molecular context-dependent dual function of miR-146a-5p, especially when the cancer grade and stage are concerned (Yuan et al., 2020). In our work, restricted by the sample size and insufficient patient characterization, we could not distinguish either the correlation between miR-146a-5p levels and the clinical outcomes or the correlation between miR-146a-5p and TRAF6, although the latter was established in cell lines.

Deep stratification in large cohorts and standardization of detection technologies are essential to substantiate the value of miR-146a-5p in early diagnosis or treatment of NSCLC, very likely in tandem with other NSCLC stage-related miRNAs (Aiso et al., 2018). There also remain many gaps in the mechanistic puzzle. (1) Do the changes in TRAF6 signaling through NF-κB and AP-1 (Walsh et al., 2015) which directly determine the downstream transcriptional events and form a feedback loop to influence miR-146a-5p expression? Being transcriptionally regulated by NF-κB, miR-146a-5p itself is an active member participating in the NF-κB-regulated networks controlling inflammatory immune responses and tumorigenesis (Yang and Wang, 2016; Mann et al., 2017). (2) Do the direct overexpression of TRAF6 and the indirect restoration of TRAF6 signaling by miR-146a-5p inhibition instigate the same downstream anti-tumor pathway in NSCLC? In an *in vivo* miR-146a^–/–^ mouse model, lowering TRAF6 expression and thereby dampening NF-κB signaling rescued functions of miR-146a-5p in myeloproliferative and pro-inflammatory regulation, but not in haematopoietic stem cell (HSC) homeostasis (Boldin et al., 2011; Magilnick et al., 2017). A plausible scenario is that miR-146a-5p and TRAF6/NF-κB may not form a single linear relationship in NSCLC development. (3) Although a strong correlation between miRNA expression in tissues and body fluids was confirmed in healthy samples, potential discordance between tissue and serum miRNA in the context of cancer is not systematically studied (Cui and Cui, 2020). As miR-146a-5p is critical in maintaining immune homeostasis by braking NF-κB-induced pro-inflammatory reaction, one of the pillar mechanisms promoting malignant transformation, the circulating miR-146a-5p in NSCLC may not only be secreted from cancer tissues but also reflect the corresponding immune activities against the specific pathological situation (Boldin et al., 2011).

Validation of miRNA as a cancer biomarker could have two different values: early diagnosis, as discussed above, and therapeutic targeting. For instance, miRNAs regulated chemo-sensitivity in NSCLC treatment (Zhang Y. et al., 2014) and miR-146a promoted the chemosensitivity to cisplatin by targeting cyclin J (Shi et al., 2017) or JNK-2 (Pang et al., 2017) in NSCLC. Targeting EGFR by EGFR- tyrosine kinase inhibitors (TKIs) has opened a new avenue in cancer therapy, as EGFR mutation frequently occurs in a large number of cancers including NSCLC. EGFR is also a target of miR-146, according to miRbase (Nicholson et al., 2001; Griffiths-Jones et al., 2008; Xu et al., 2012). The miR-146 level was found to have a positive predictive value for patients’ response to TKIs in NSCLC treatment (Leonetti et al., 2019). Although only certain cancer cell phenotypes were examined, the present work showed that a simple intervention of TRAF6 was sufficient to reverse the malignant behavior of the NSCLC cell lines.

Taken together, despite the discrepancy in the literature, we have placed a diagnostic and therapeutic value on miR-146a-5p and its direct target TRAF6 as part of the molecular signature marking NSCLC development. To translate TRAF6-dependent miR-146a-5p regulation into clinical application, in a cancerous niche subject to the influence of the immune system and other microenvironmental factors, further analysis of large-scale clinical samples is imperative.

## Data Availability Statement

All datasets generated for this study are included in the article/Supplementary Material.

## Ethics Statement

The studies involving human participants were reviewed and approved by Ethics Committee of Second Affiliated Hospital of Dalian Medical University. The patients/participants provided their written informed consent to participate in this study.

## Author Contributions

YW, QW, LH, and XL conceived the study, designed the experiments, and wrote the manuscript. XL, BoL, FW, and RL performed the experiments, contributed to the collection of samples, clinical data, and analyzation of data. MZ, YB, JW, NW, DH, and RL also performed some of the experiments, analyzed data, and involved in discussion. ZL, BF, GZ, SW, LZ, JM, YY, and BiL also designed the experiments, involved in discussion, and wrote the manuscript. All authors reviewed the manuscript.

## Conflict of Interest

The authors declare that the research was conducted in the absence of any commercial or financial relationships that could be construed as a potential conflict of interest.

## Footnotes

1 https://www.cancer.gov
