# Supplementary figures and images for: miR-146a-5p Plays an Oncogenic Role in NSCLC via Suppression of TRAF6
Source: Front Cell Dev Biol. 2020 Sep 2;8:847. doi: 10.3389/fcell.2020.00847 (PMC7493784; doi:10.3389/fcell.2020.00847)

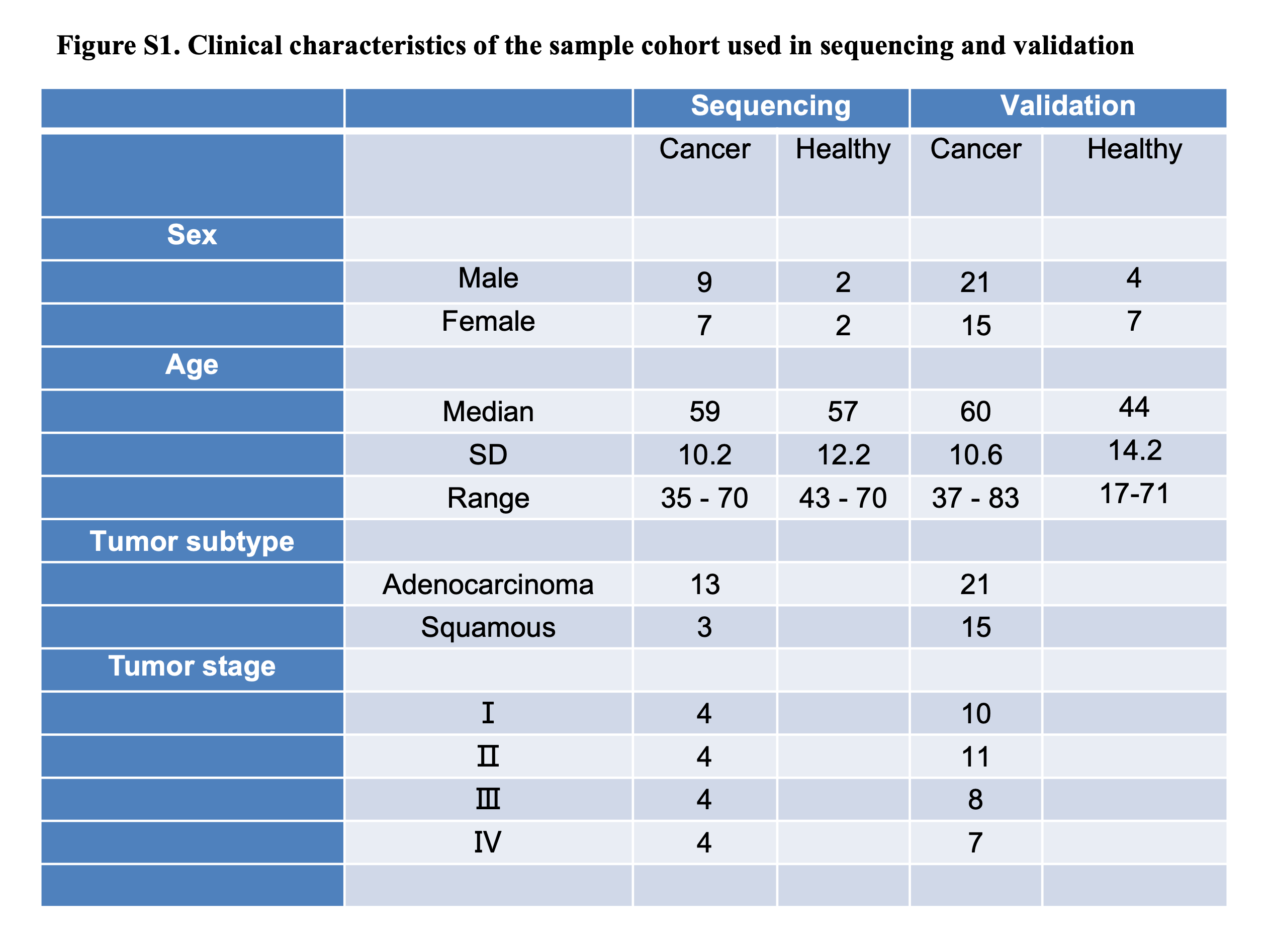

Supplement: Supplementary file 1 [file Image_1.tiff]

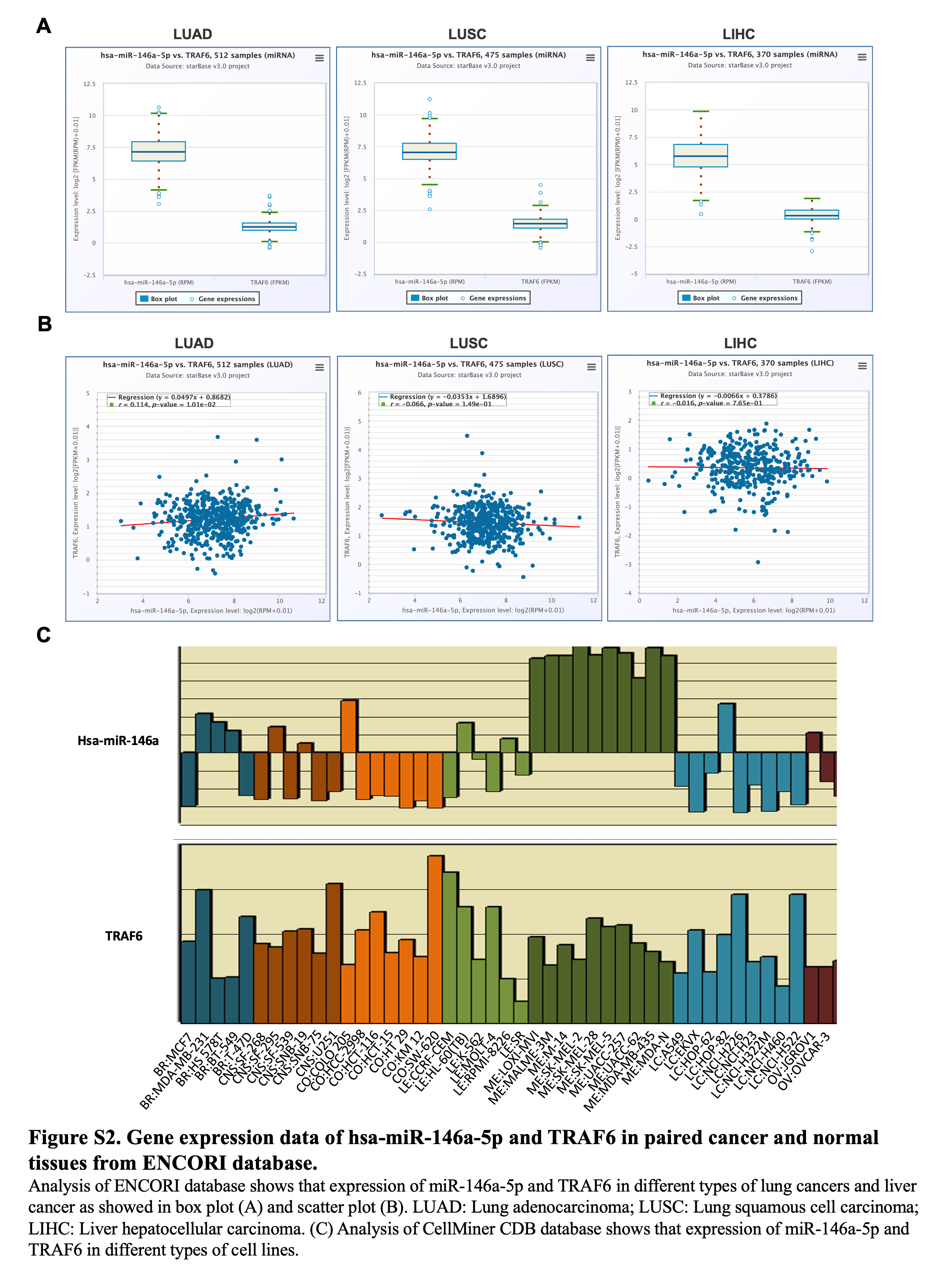

Supplement: Supplementary file 2 [file Image_2.tiff]
