# Supplementary material for: miR-146a-5p Plays an Oncogenic Role in NSCLC via Suppression of TRAF6
Source: Front Cell Dev Biol. 2020 Sep 2;8:847. doi: 10.3389/fcell.2020.00847 (PMC7493784; doi:10.3389/fcell.2020.00847)
Supplement: Supplementary file 3 [file Table_1.pdf]

Table S1. Sequences of RNA and DNA oligonucleotides used in transfection and cloning

**RNA oligonucleotides**		**Sequence**
miR-146a-5p mimic	Sense	5′-UGAGAACUGAAUUCCAUGGGUU-3′
	Antisense	5′-CCCAUGGAAUUCAGUUCUCAUU-3′
miR-146a-5p mimic control	Sense	5′-UUCUCCGAACGUGUCACGUTT-3′
	Antisense	5′-ACGUGACACGUUCGGAGAATT-3′
miR-146a-5p inhibitor	Sense	5′-AACCCAUGGAAUUCAGUUCUCA-3′
miR-146a-5p negative control inhibitor	Sense	5′-CAGUACUUUUGUGUAGUACAA-3′
siTRAF6-1	Sense	5′-GGGUACAAUACGCCUUACATT-3′
	Antisense	5′-UGUAAGGCGUAUUGUACCCTT-3′
siTRAF6-2	Sense	5′-GCAGUGCAAUGGAAUUUAUTT-3′
	Antisense	5′-AUAAAUUCCAUUGCACUGCTT-3′
siRNA negative control	Sense	5′-UUCUCCGAACGUGUCACGUTT-3′
	Antisense	5′-ACGUGACACGUUCGGAGAATT-3′
pmirGLO-TRAF6-WT	Sense	5′-CGGGCCCTATAGATATAAAATATCGTGGAATCTAGTTCTCAGGGAGACCC-3′
	Antisense	5′-TCGAGGGGTCTCCCTGAGAACTAGATTCCACGATATTTTATATCTATAGGGCCCGAGCT-3′
pmiRGLO-TRAF6-MUT	Sense	5′-CGGGCCCTATAGATATAAAATATCGACCTTACTAGTTCTCAGGGAGACCC-3′
	Antisense	5′-TCGAGGGGTCTCCCTGAGAACTAGATAGGACATATTTTATATCTATAGGGCCCGAGCT-3′
