# Supplementary material for: miR-146a-5p Plays an Oncogenic Role in NSCLC via Suppression of TRAF6
Source: Front Cell Dev Biol. 2020 Sep 2;8:847. doi: 10.3389/fcell.2020.00847 (PMC7493784; doi:10.3389/fcell.2020.00847)
Supplement: Supplementary file 4 [file Table_2.pdf]

Table S2. Primer sequences for quantitative RT-PCR

Gene		Primer Sequence
miR-146a-5p	Forward	5′-UGAGAACUGAAUUCCAUGGGUU-3′
	Reverse	5′-ACTCTTGACTTAAGGT ACCCAA-3′
TRAF6	Forward	5′-ATGCGGCCATAGGTTCTGC-3′
	Reverse	5′-TCCTCAAGATGTCTCAGTTCCAT-3′
U6	Forward	5′-CTCGCTTCGGCAGCACA-3′
	Reverse	5′-AACGCTTCACGAATTTGCGT-3′
GAPDH	Forward	5′-AATGGATTTGGACGCATTGGT-3′
	Reverse	5′-TTTGCACTGGTACGTGTTGAT-3′
