# Supplementary material for: miR-146a-5p Plays an Oncogenic Role in NSCLC via Suppression of TRAF6
Source: Front Cell Dev Biol. 2020 Sep 2;8:847. doi: 10.3389/fcell.2020.00847 (PMC7493784; doi:10.3389/fcell.2020.00847)
Supplement: Supplementary file 5 [file Table_3.pdf]

Table S3. Information of primary antibodies for Western blot

Name	Supplier	Dilution rate
TRAF6	Proteintech	1:1000
BCL-2	Abbkine	1:1000
BAX	Abbkine	1:1000
Cleaved caspase 3	Elabscience	1:500
Caspase 3	Abbkine	1:1000
NF-kB-p65	Proteintech	1:1000
NF-κB-p65(pS536)	Active Motif	1:500
PARP	Cell signaling Technology	1:1000
β-actin	Proteintech	1:2000
GAPDH	Proteintech	1:1000
